# Lower Limb Unilateral and Bilateral Strength Asymmetry in High-Level Male Senior and Professional Football Players

**DOI:** 10.3390/healthcare11111579

**Published:** 2023-05-28

**Authors:** Mário C. Espada, Marco Jardim, Rafael Assunção, Alexandre Estaca, Cátia C. Ferreira, Dalton M. Pessôa Filho, Carlos E. L. Verardi, José M. Gamonales, Fernando J. Santos

**Affiliations:** 1 Instituto Politécnico de Setúbal, Escola Superior de Educação, 2914-504 Setúbal, Portugal; catia.ferreira@ese.ips.pt (C.C.F.);; 2 Life Quality Research Centre, Complexo Andaluz, Avenida Dr. Mário Soares 110, 2040-413 Rio Maior, Portugal; 3 CIPER, Faculdade de Motricidade Humana, Universidade de Lisboa, 1499-002 Lisboa, Portugal; 4 Instituto Politécnico de Setúbal, Escola Superior de Saúde, 2914-503 Setúbal, Portugal; marco.jardim@ess.ips.pt (M.J.); rafael.assuncao@ess.ips.pt (R.A.); 5 Casa Pia Atlético Clube, Estádio Pina Manique, Parque de Monsanto, 1500-462 Lisboa, Portugal; alexandre.estaca@gmail.com; 6 Research Group in Optimization of Training and Performance Sports, Faculty of Sport Science, University of Extremadura, 10005 Cáceres, Spain; martingamonales@unex.es; 7 Department of Physical Education, São Paulo State University (UNESP), Bauru 17033-360, Brazil; dalton.pessoa-filho@unesp.br (D.M.P.F.); carlos.verardi@unesp.br (C.E.L.V.); 8 Graduate Programme in Human Development and Technology, São Paulo State University (UNESP), Rio Claro 13506-900, Brazil; 9 Graduate Programme in Developmental Psychology and Learning, Faculty of Science, São Paulo State University (UNESP), Bauru 17033-360, Brazil; 10 Faculty of Health Sciences, University of Francisco de Vitoria, 28223 Madrid, Spain; 11 Faculdade de Motricidade Humana, Universidade de Lisboa, 1499-002 Lisboa, Portugal

**Keywords:** soccer, strength, interlimb, symmetry, injury risk, performance

## Abstract

This study sought to assess the relationship between different jumping asymmetries and associated performance variables in high-level male senior and professional football players. Nineteen football players with at least 12 years of training experience (23.2 ± 3.1 years of age; 75.2 ± 4.8 kg of body mass and 181 ± 0.06 cm of height) participated in this study performing countermovement jump (CMJ), squat jump (SJ), single-leg CMJ and drop jump (DJ), associated performance variable eccentric utilization ratio (EUR), stretch-shortening cycle (SSC), bilateral deficit (BLD), and limb symmetry index (LSI) were determined. High correlations were observed between different methodologies of jump tests and associated performance indicators (SSC, BLD, EUR), except LSI. Moreover, CMJ and SJ results were different (*p* < 0.05), but no differences were found between interlimb in CMJ (*p* = 0.19) and DJ (*p* = 0.14). Between the same limbs and different jumps differences were detected in CMJ and DJ (*p* < 0.01), and it has also been found that the laterality effect size on strength was small in CMJ (*ES* = 0.30) and DJ (*ES* = 0.35). LSI between CMJ and DJ was not different despite higher mean values in CMJ, and although mean BLD was positive (>100%), the results highlight the need for individual evaluation since eight players scored negatively. An in-depth and accurate analysis of performance in preseason screening jump tests should be considered, aiming to detect injury risk, specifically evaluating different jumping test methodologies, and determining jumping associated performance variables for each test, namely EUR, SSC, BLD, and LSI. Specific muscle-strengthening exercises could be implemented based on this study results and outcomes, aiming to reduce injury risks and lower extremity asymmetries and to enhance individual football performance in high-level male senior and professional football players. Sports institutions should pay special attention regarding potential health problems in athletes exposed to daily high training loads.

## 1. Introduction

Football is one of the most popular sports worldwide [1], characterized by a variety of unpredictable movements involving rapid speed changes [2]. According to previous studies, this sport is associated with a higher injury rate compared to other competitive sports since the incidence of injury is estimated to be 15 to 35 injuries per 1000 h of training/matches [3,4], and specifically, muscle injuries (>90%) occur in noncontact situations [5,6], indications that are associated with a special care in training evaluation and prescription and monitoring of the competitive events.

Interlimb asymmetry (or bilateral difference, bilateral asymmetry), defined as differences between the dominant and non-dominant limb function or performance [7], may be related to long-term training in the same sport [8] and has been a popular source of investigation in recent years. Playing football demands fast and powerful movements, implying lower-limb muscles in maximal and rapid actions [9]. In football, players are exposed to an increasing number of competitive events and high volumes of regular training, associated with a preferred limb dominance [10,11]. Functional asymmetric adaptations may therefore relate to the asymmetric motor demands [12] which characterize particular sports [13].

The repetition of execution of unilateral movements or skills is one of the particularities of football in training and competitive events (e.g., cutting, changing direction, and kicking) [14]; consequently, the increased interlimb asymmetry may be important considering injury risks under the fatigued state [15]. A minimum symmetry target of 10% has been previously suggested for evaluation and rehabilitation protocols [16], being equally considered for clearance to return to practice after anterior cruciate ligament reconstruction (ACLR) [17]. Lower limb injuries are a serious issue in professional athletes because they may be associated with long-term absence from match and health-related problems and related to decreased performance and financial costs [18]. On average, for example, typical hamstring strain injuries causes athletes to miss 2 weeks of training or competitive play [19], with this timeline potentially representing up to 6 fixtures being missed, with professional football teams often experiencing 5–6 hamstring injuries per season [5].

Strength is a very important physical quality in football that underpins successful performance in the complex and varied motor skill tasks in this sport [20,21]. Jump tests are a viable method of quantifying interlimb asymmetries and have successfully been used to prospectively identify the risk of injury of athletes [22]. The countermovement jump (CMJ) and squat jump (SJ) are among the most regularly used vertical jumps for evaluation purposes [23,24], and drop jump (DJ) is frequently used as a plyometric exercise to develop and evaluate jumping performance [25]. The performance in CMJ (height) is on average greater than the height of the SJ [24,26], and the difference between the jumps, often indicated as the eccentric utilization ratio (EUR), has been suggested as an indicator of performance [26]. Lower values of EUR denote that the athlete should improve elasticity storage, which is usually developed with explosive exercises; in contrast, athletes with higher EUR are normally directed to training basic strength.

Associated with CMJ and SJ is also the stretch-shortening cycle (SSC), previously indicated as a stretching of the muscle–tendon unit prior to a shortening [27] and linked to increased force, mechanical work, torque, and power during the shortening phase of the SSC [28,29]. More specifically, during CMJ, bilateral deficit (BLD) has been discussed, although the elements that influence the BLD are not well defined in the literature [30]. Recently, Pleša et al. [31] stressed that higher BLD could reflect improved neuromuscular capacity in unilateral tasks compared with the bilateral and found that larger a BLD is associated with superior linear sprint and approach jump performance in volleyball players, suggesting BLD as a tool to assist practitioners in decision making in athletic training. A score below 100% is indicative of a bilateral strength deficit, while a bilateral index higher than 100% is indicative of bilateral facilitation (BLF), where the force of both legs during a bilateral exercise exceeds the sum of the unilateral movements.

Football demands explosive movement performance such as change of direction speed, heading, and shooting [32], and all these are often associated with single-leg movements at some point. The limb symmetry index (LSI) is associated with single-leg tests and considered, for example, in return-to-sport decision making for individuals after ACLR [17,33]. It was previously reported by Krych et al. [34] that 6 months after traditional ACLR, younger age was a key factor associated with exceptional strength and functional recovery (defined as strength LSI > 85%), and previous research highlighted that muscle function tests are strong determinants for between-limb asymmetry predictions in ACLR [35].

Even though some level of asymmetry is probable in football players, few investigators have examined the prevalence of asymmetry during commonly used screening tests in high-level football players, mainly because they are exposed to intense training and competitive schedules. It was previously indicated that asymmetries measured during common screening protocols may be associated with reductions in jumping performance [14] and that it is important to identify reliable methods of assessing the direction and magnitude of asymmetries to reduce the potential risk for injury and re-injury in all populations [19]. To the authors’ knowledge, to date, no study with high-level male senior and professional football players has compared different jumping methods, considering unilateral and bilateral perspectives and the respective associated performance variables (EUR, SSC, BLD, and LSI), which may provide relevant information regarding evaluation purposes and specific intervention procedures aiming injury prevention and performance enhancement. Hence, the aim of the present study was to assess the relationship between different jumping asymmetries and associated performance variables in high-level male senior and professional football players.

## 2. Materials and Methods

### 2.1. Subjects

Nineteen football players with at least 12 years of training experience (23.2 ± 3.1 years of age; 75.2 ± 4.8 kg of body mass and 181 ± 0.06 cm of height) were involved in this research. All subjects, integrated in a Portuguese first division football team, trained for at least 36 weeks per year with regular 5 training sessions per week (3–4 h per day), plus 1 or 2 competitive events a week, in the preceding 12 months before participation in the research. The criteria for not participating in the study were injury associated with absence from training more than 4 days in the 3 months before the research and injury related to surgical intervention in the 12 months before data collection.

International ethical standards for sport and exercise science research [36] were followed and the study also considered the Declaration of Helsinki. The Ethical Committee of the Polytechnic Institute of Leiria approved the research (CE/IPLEIRIA/22/2021) and club authorization was obtained before contact with the athletes, with all of these providing written informed consent. No economic incentives were provided, and goalkeepers were excluded from this study because of their different training and physical characteristics. Participants and the clubs’ medical staffs were informed about the potential risks of the current procedures and provided written informed consent.

### 2.2. Study Design

Subjects were tested on two occasions at the same time of day to avoid circadian rhythm effects, each separated by 24 h. All footballers arrived at the club’s training facility wearing appropriate footwear and comfortable athletic clothing and refrained from intense training at least 48 h prior to testing.

Previous to data collection, no specific familiarization procedures were necessary because athletes were all experienced players, many of them with national team international participation in their national countries, with regular involvement in strength training and evaluation events. ChronoJump photoelectric cells (Bosco System, Barcelona, Spain) was used to evaluate the jump´s performance and a 30 cm step platform (Reebok^®^) during the DJ. The height of the athletes was evaluated on a measurement platform (Seca 274, Milan, Italy) and body mass (kg) determined on a calibrated physician scale (Seca 786 Culta, Milan, Italy).

### 2.3. Procedures

A dynamic 20 min warm-up was performed before data collection, and afterward all subjects practiced each jump test as many times as they wanted, although all players were instructed to practice each test a minimum of three times.

A 3 min rest period was prescribed between the end of warm-up and the first test. After that, athletes performed the tests, simultaneously assessed by two experienced observers with doctoral degrees in sports sciences, accompanied in the data collection by 3 members of the club’s clinical staff. A total of three attempts were completed in all jumps, and observers were blinded to each other’s scores. The higher value was considered for the final analysis [30], and a 1 min rest was granted between each jump to minimize the effect of fatigue [31].

During testing, no motivational communication took place because communication is a relevant element in the coach–athlete relationship [37] and could have influence the performance and score.

### 2.4. Testing Description

#### 2.4.1. Squat Jump (SJ)

Subjects begin by standing on the designated testing leg with their hands on hips. Subjects were then instructed to perform with knees bent in a 90° flexion position and afterward, after a short pause moment, and jump as far forward as possible, landing on both legs. Upon landing, the players were required to ‘hold and stick’ their position for 2 s. Failure to stick the landing resulted in a void trial and the jump being retaken after a 60 s rest. This was a criterion for all jump tests.

#### 2.4.2. Countermovement Jump (CMJ)

Participants begin by standing with their hands on hips and were instructed to start from the standing position and use an explosive countermovement (until the 90° knee flexion position), jumping as high as possible.

#### 2.4.3. Single-Leg Countermovement Jump (SL-CMJ)

Subjects paused in an upright position with hands on hips and feet positioned hip-width apart. To start the test, one leg was lifted off the floor to approximately mid-shin-height of the standing leg. Subjects afterward performed a countermovement to 90° flexion position, followed by a quick upward vertical movement, triple extending at the ankle, knee, and hip, with the objective of jumping as high as possible. The jumping leg had to remain fully extended, and hands fixed to hips; any variation from this required the trial be re-taken after a 60 s rest period [22].

#### 2.4.4. Single-Leg Drop Jump (SL-DJ)

Football players were asked to perform single-leg drop jump (SL-DJ) trials on both legs after hopping from a box of 30 cm height positioned 5 cm posterior to the ChronoJump photoelectric cells. It was requested that the participants take off standing on a single-leg, land on the same leg, and stabilize as quickly as possible to afterward perform a quick upward vertical movement, as described by [38].

### 2.5. Measurments

#### 2.5.1. Limb Asymmetry Index (LSI)

The asymmetry index was calculated using the equation (dominant limb–non-dominant limb)/dominant limb) × 100 [39].

#### 2.5.2. Bilateral Deficit (BLD)

The BLD was computed according to the following formula [40]:

BLD (%) = [100 × (Bilateral/Right unilateral + Left unilateral)] − 100

#### 2.5.3. Eccentric Utilization Ratio (EUR)

The EUR was calculated as the ratio between CMJ and SJ heights and considered an indicator of lower extremity SSC performance in athletes. An ideal EUR of ~1.1 was previously suggested, in which the CMJ score should be 1.1× (10%) that of the SJ [26].

#### 2.5.4. Stretch-Shortening Cycle (SSC)

The theory behind SSC is that the muscles and tendons are able to store elastic energy in the pre-stretch phase of the movement. One example of this is a CMJ, which tendentially produces more force than an SJ. For SSC measurement, the difference between the two types of vertical jumps is used (CMJ-SJ) [41].

### 2.6. Statistical Analysis

Previous to data collection, the sample size required was determined (GPower, v.3.1.9, University of Kiel, Kiel, Germany). The sample was deemed as adequate based on a statistical power of 75%, effect size (5% change), and α error probability of 0.05 associated with 18 subjects. Data was initially organized as means and standard deviations (M ± SD) in Microsoft Excel™; all additional analyses were made in Statistical Package for Social Sciences v27.0 (SPSS, IBM Corp, Armonk, NY, USA). Reliability of tests measures was estimated using intraclass correlation coefficient (ICC) with absolute agreement and 95% confidence intervals. Interpretation considered previous research [42] specifically values > 0.9 = excellent, 0.75–0.9 = good, 0.5–0.75 = moderate, and <0.5 = poor.

The linear regression models between different test results were computed, the trendline equations and determination coefficients (R^2^) were calculated. Cohen’s d was used for effect size (*ES*) calculation and considered extremally large (>4), very large (2–4), large (1.2–2), moderate (0.6–1.2), small (0.2–0.6), and trivial (0–0.2) [43].

Pearson’s r correlation coefficients between assessment methods were determined, with the absolute value of the correlation demarcated as follows [44]: negligible correlation (r^2^ < 30), weak correlation (r^2^ = 0.30–0.50), moderate correlation (r^2^ = 0.50–0.70), strong correlation (r^2^ = 0.70–0.90), and very strong correlation (r^2^ > 90). Paired-sample t-test was used to compare the studied variables and data obtained with both lower limbs (left and right). Statistical significance was accepted at *p* ≤ 0.05.

## 3. Results

High levels of reliability (ICC > 0.87 and <0.94) were observed in all the test results and studied variables, with the lower levels associated with single-leg evaluations. Descriptive statistics of all tests results are shown in Table 1.

Results associated with CMJ and DJ were different (*p* < 0.01). No statistically significant differences were determined between the right and left leg in the CMJ (*p* = 0.19) and DJ (*p* = 0.14) contrary to the same single-leg performance in both CMJ and SJ. It has also been found that the laterality effect size on strength was small in CMJ (*ES* = 0.30) and DJ (*ES* = 0.35). Moreover, despite the mean value of SSC being positive, two players exhibited negative values, −3.37 and −3.13, which represented as consequence in these two specific cases a EUR value of 0.97, the minimum observed.

Considering LSI and the cut-off threshold of 85%, it should be highlighted that it was detected in five players an LSI-DJ below 85%, while regarding LSI-CMJ, only two presented values below this boundary threshold. Regarding BLD, eight players scored lower values than 100%, indicative of bilateral strength deficit, while the other eleven cohorts, exhibited BLF (BLD > 100%), since the force of both legs during a bilateral exercise exceeds the sum of the unilateral movements. Table 2 shows the correlations between the studied variables.

**Figure 1:**
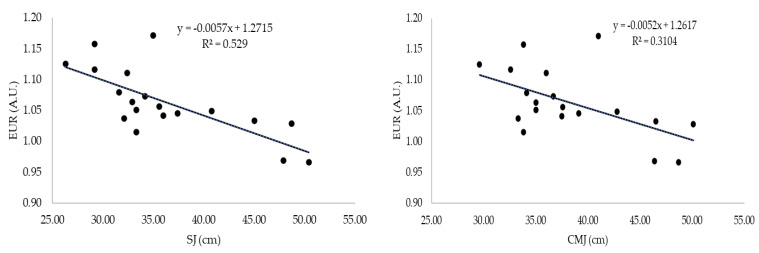
Linear regression of squat jump and countermovement jump on eccentric utilization ratio.

Several correlations were visible between performance in jumping tests and associated performance variables (EUR, SSC, BLD), although no correlations were observed between LSI-CMJ, LSI-DJ, and all the other studied variables. Figure 1 depicts the linear regression of SJ and CMJ on EUR.

## 4. Discussion

The aim of the present study was to assess the relationship between different jumping asymmetries and associated performance variables in high-level male senior and professional football players. The main findings were: (1) high correlations were observed between different methodologies of jump tests (the execution dynamic of CMJ is different compared to SJ) and associated performance indicators (SSC, BLD, EUR), except LSI; (2) CMJ and SJ results were different and should not be used solely and interchangeably as screening tests performed during preseason to evaluate players physical condition; (3) no differences were found between interlimb in CMJ and DJ, although between the same limbs and different jumps differences were detected; (4) LSI between CMJ and DJ were not different, despite higher mean values in CMJ and; (5) although mean BLD was positive (>100%), the results highlight the need for individual evaluation in screening tests performed during preseason in high-level male senior and professional football since eight players scored negatively, which is also underlined by the maximum, minimum and standard deviation of the results in the present study.

Asymmetry was indicated as a potential risk factor for injury in football players [45] that may be the result of additional stress placed on the soft tissue structures of the nondominant leg [46]. Previously, musculoskeletal imbalances of 10% on at least one jump test were observed in male youth football players [11]. Later, Lockie et al. [47] reported larger interlimb differences for the SL-CMJ (10.4%) in male team sport athletes aged 23 years, and more recently, asymmetries of 15% during landing tasks were shown in uninjured 10–18-year-old elite male football players [45]. Additionally, Bishop et al. [48] conducted a study with elite youth female football players and reported significantly larger asymmetries for SL-CMJ (12.5%). Similar observations were previously shown, reporting larger asymmetry for SL-CMJ (11.2%) in adolescent male handball players aged 16 years [49].

In the present study, CMJ and SJ results were different, and no differences were observed between interlimb in CMJ and DJ, more specifically, CMJ (*p* = 0.19) and DJ (*p* = 0.14). Although different jump differences were found between the same limbs and the laterality effect size on strength was small in CMJ (*ES* = 0.30) and DJ (*ES* = 0.35), these results lead us to suggest that CMJ and SJ should not be used solely and interchangeably as screening tests performed during preseason to evaluate physical condition of high-level male senior and professional footballers. Moreover, biceps femoris and soleus may impact jump performance because an early activation of the biceps femoris has been found to negatively influence the joint power transfer [50], consequently reducing the effect of the SSC, a key factor related to performance in vertical jumps [51], which allows us to speculate that CMJ interlimb asymmetry may be particularly related with lateral asymmetry of these muscles.

The associated mechanisms associated with SSC are highly debated in the literature as none of the mechanisms can completely explain this SSC-effect [52,53]. The preactivation of the muscle [54], the stretch-reflex [55], and the release of stored passive-elastic energy in the tendinous tissue [56,57] are mechanisms attributed to this effect. In this study, despite the mean value of SSC being positive, two players exhibited negative values, −3.37 and −3.13, which represented as a consequence in these two specific cases an EUR value of 0.97. This evidence highlights the importance of analyzing not only different methodology and performance of jump tests but also associated performance such as SCC, and very importantly, bearing in mind an individual perspective since football team mean values do not represent individual potential injury risk.

The EUR is used as an indicator of elastic storage in CMJ in strength and conditioning practice [58]. Larger EUR can be associated with superior CMJ performance but may also lower SJ performance. Moreover, poor SJ performance could be related to high levels of muscle slack [24] or to poor ability to develop force rapidly [59]. This is supported by the evidence that individuals with stiffer tendons, a trait that is beneficial for rapid force development, exhibit lower EUR [60], and it should also be noted that EUR was larger in track and field athletes compared to gymnasts and parkour practitioners, while the jumping performance (SJ and CMJ) was better in the latter cases compared to that in track and field athletes [61]. In the present study, we found no correlations between EUR and other jump tests associated performance variables, namely BDL and LSI, only correlations with SJ (r = −0.74, *p* < 0.01) and CMJ (r = −0.57, *p* < 0.05); however, r-squared of the regression analysis between EUR and both jump methodologies was not very strong, confirming the evidence that the utilization of stored elastic energy plays a short role in CMJ, except when executed with low amplitude [62].

The BLD phenomenon is related to the evidence that force production during a maximal bilateral contraction normally is lower compared to the sum of total force produced by the left and right limbs combined [63]. For this reason, true examination of the BLD is only possible when comparable bilateral and unilateral tasks have been completed (e.g., bilateral and unilateral CMJ). Previously, Bračič et al. [64] observed a BLD of 19.1% in 12 elite sprinters. and a jump height BLD of 8.5% was reported in trained volleyball players [65]. More recently, it was shown that the DJ test showed BLF contrarily to the CMJ, the authors emphasized that this may offer further support showing the advanced technical nature of reactive strength tasks [66]. Considering BLD and the results in the present study, although mean BLD was positive (>100%), we found that eight high-level male senior and professional football players scored lower than 100%, which is indicative of bilateral strength deficit, while the other eleven cohorts exhibited BLF (BLD > 100%), since the force of both legs during a bilateral exercise exceeds the sum of the unilateral movements. This evidence underlines that when left and right limbs were summed and still could not exceed the bilateral score, this demonstrates how challenging unilateral fast SSC activities are, which was previously supported in [67], research which showed that both endurance and power-based athletes showed a BLF of −3.8 to −13.8% in power during the DJ test, emphasizing that the wide variation in BLD scores in our and other studies led Bishop et al. [66] to suggest that this phenomenon should be investigated with multiple metrics for each test.

Nevertheless, absolute strength is an important factor influencing bilateral strength asymmetry [68], and the effects of strength asymmetry and/or limb preference on dynamic athletic movements such as running, jumping and kicking [69], some of the most common or routine movements in football during training and competition, were verified. In addition, it has been demonstrated that strength asymmetry of >15% caused a reduced jump height [68]; therefore, the cut-off threshold of 85% appears to be strengthened by the latter findings. In another study, Kotsifaki et al. [70] indicated that measuring only jump distance, even using the healthy leg as a reference, is not sufficient to fully assess knee function after ACLR, and Wang et al. ([71] suggested that natural mechanics could be responsible for asymmetry in able-bodied walking instead of neurophysiological mechanisms, for example, leg dominance, something that should be considered in football because the repetition in quadriceps activity can have as a consequence muscular imbalances between limbs [72].

In the present study, we found that although LSI between CMJ and DJ was not different despite higher mean values in CMJ, in five high-level male senior and professional football players an LSI-DJ below 85% was observed, while regarding LSI-CMJ, only two presented values were below this threshold boundary. Moreover, no correlations were noted between LSI-CMJ, LSI-DJ, and all the jumping associated performance variables. Considering this context, it must be mentioned that when the dominant leg of football players is associated with dynamic movements and powerful contractions, such as shooting the ball, the contralateral non-dominant leg is solicited in a different way by ensuring body stability related to isometric contractions. This motor mechanism repeatedly performed during training and competition could explain some of the differences between both limbs in asymmetrical sports. Noteworthy, the maximum, minimum, and standard deviation results found in our study suggest the need for individualized analysis in screening tests performed during preseason and also individualized training prescription and recovery methods in high-level male senior and professional football players.

Thus, from a practical applications perspective, it seems prudent to suggest that strength training should be considered in high-level male senior and professional football players, as recently suggested for football academy players [73,74]. Finally, football club structures (in concrete, some of those associated with decision making regarding daily training conditions) should assume that collaborative work developed by professionals of different areas is a plus for athlete’s global condition and may represent an important support in the definition of evaluation procedures, specifically protocols aiming to improve injury prevention and, at the same time, to improve sports performance [18].

It is important to note a few limitations within this study: (a) The possibility that the results cannot be directly considered and applied to other, age categories, gender or level football players, because in this study participated male high-level male senior and professional football players, with experiences and training routines that do not characterize the vast majority of football players. (b) The study was conducted during the preseason (August); consequently, the protocols and results should not be considered and applicable in other moments such during high intensity training load (for example in training camps without competition events and double or triple training sessions) or weeks with several competitive events (weekends and mid-week).

Future studies should consider intervention strategies to effectively focus the interlimb asymmetry concern. It is also advisable to increase the sample size and expand data collection in more teams of the same football league with different training methodologies or even other-level teams of different leagues. We also suggest the replication of this study with other age-categories, gender, and in different events of the season or even longitudinally between seasons, including the possibility of injury occurrence analysis.

## 5. Conclusions

The findings of the present study emphasize that high-level male senior and professional football players are characterized by fairly good mean values of lower limb symmetry. However, it should be noted that an in-depth and accurate analysis of jumping performance in preseason screening tests should be considered aiming to detect injury risk and specifically evaluating different jumping test methodologies and determining jumping associated performance variables for each test, namely EUR, SSC, BLD, and LSI.

The correlations between most of the strength tests and jumping-associated performance variables underline the relationship between these, which should be considered for physical evaluation, in a longitudinal tracking performance during football season, and in a pluriannual perspective, and injury prevention and performance enhancement in football programs. From a training perspective, it should be noted that bilateral training should in no way be ignored, although unilateral strength exercises will aid in the development of unilateral movement competency.

The results in this study highlight that screening for muscle strength and asymmetry could be of particular importance for football injury prevention, and sports institutions should pay special attention regarding potential health problems in athletes exposed to daily high training loads. Specific muscle-strengthening exercises could be implemented based on this study’s results and outcomes, aiming to reduce injury risks and lower extremity asymmetries and to enhance individual football performance in high-level male senior and professional football players.

## Figures and Tables

**Table 1:** Mean ± standard deviation (M ± SD), minimum and maximum of performance in all tests.

Variables	Mean	SD	Minimum	Maximum
SJ (cm)	36.38 *	6.98	26.30	50.40
CMJ (cm)	38.40 *	5.92	29.60	50.10
EUR (A.U.)	1.06	0.06	0.97	1.17
SSC (%)	6.27	5.50	−3.37	17.14
SL-CMJ (L) (cm)	20.68 ^#^	4.73	12.70	30.90
SL-CMJ (R) (cm)	21.29 **	5.10	14.80	35.00
LSI-CMJ (A.U.)	92.57	4.98	83.13	99.44
BLD-CMJ (A.U)	11.71	27.68	−33.32	70.22
SL-DJ (L) (cm)	23.41 ^#^	4.89	16.90	31.50
SL-DJ (R) (cm)	24.64 **	5.36	17.80	33.80
LSI-DJ (A.U.)	89.06	7.79	70.03	97.78

M, Mean; SD, Standard deviation; SJ, Squat jump; CMJ, Countermovement jump; EUR, Eccentric utilization ratio; SSC, Stretch-shortening cycle; SL-CMJ (L), Single-leg countermovement jump (left); SL-CMJ (R), Single-leg countermovement jump (right); LSI-CMJ, Limb symmetry index—countermovement jump; BLD-CMJ, Bilateral deficit countermovement jump; SL-DJ (L), Single-leg drop jump (left); SL-DJ (R), Single-leg-drop jump (right); LSI-DJ, Limb symmetry index—drop jump; * and ^#^ significant difference (*p* < 0.01); ** significant difference (*p* < 0.05).

**Table 2:** Correlations between the studied variables associated with jumps.

	CMJ	EUR	SSC	SL-CMJ (L)	SL-CMJ (R)	BDL-CMJ	SL-DJ (L)	SL-DJ (R)
SJ	0.97 **	−0.74 **	−0.73 **	0.71**	0.66 **	-	0.59 **	0.60 **
CMJ		−057 *	−0.56	0.73 **	0.65 **	-	0.59 **	0.59 **
EUR			0.99 **	-	-	-	-	-
SSC			-	-	-	-	-	-
SL-CMJ (L)				-	0.92 **	−0.67 **	0.79 **	0.79 **
SL-CMJ (R)					-	−0.57 *	0.71 **	0.71 **
BDL-CMJ						-	−0.55 *	−0.55 *
SL-DJ (L)							0.77 **	0.99 **
SL-DJ (R)								1

SJ, Squat jump; CMJ, Countermovement jump; EUR, Eccentric utilization ratio; SSC, Stretch-shortening cycle; SL-CMJ (L), Single-leg countermovement jump (left); SL-CMJ (R), Single-leg-countermovement jump (right); BLD-CMJ, Bilateral deficit countermovement jump; SL-DJ (L), Single-leg drop jump (left); SL-DJ (R), Single-leg drop jump (right). * (*p* < 0.05), ** (*p* < 0.01).

## Data Availability

The data that support the findings of this study are available from the corresponding and first authors (mario.espada@ese.ips.pt and fernando.santos@ese.ips.pt) upon reasonable request.
